# Escaping the Ice Age: The Evolution of Kidney Preservation from Cold Storage to Normothermic Perfusion

**DOI:** 10.34067/KID.0000001147

**Published:** 2026-03-26

**Authors:** Scott G. Westphal, Shaheed Merani

**Affiliations:** 1 Department of Internal Medicine, University of Nebraska Medical Center, Omaha, Nebraska; 2 Department of Surgery, University of Nebraska Medical Center, Omaha, Nebraska

**Keywords:** clinical nephrology, immunosuppression, renal transplantation, transplant outcomes, transplantation

Kidney transplantation is the optimal treatment for ESKD, but a persistent shortage of organs suitable for transplant has created a continual supply and demand mismatch. Initiatives to expand the donor pool and reduce discards have been a forefront priority of the transplant community, providing impetus to consider kidneys that were historically not used for transplant, including kidneys from donors with older age or comorbid conditions, and increased utilization of kidneys from donors after circulatory death. These initiatives have led to a steady increase in the annual number of US deceased donor kidney transplants. Yet, kidney discard rates remain high, particularly donors after circulatory death kidneys, due to concerns about warm ischemia time resulting in ischemia-reperfusion injury, which manifests clinically as delayed graft function (DGF) or primary nonfunction.

As more “marginal” kidneys are considered for transplant, there is greater need for reliable tools to both rehabilitate and prognosticate donor kidneys before transplant. Despite its use in organ allocation systems, the Kidney Donor Profile Index has only modest capacity to predict post-transplant outcomes (c-statistic approximately 0.6).^1^ Kidney histology, although commonly obtained, also does not reliably predict post-transplant outcomes. Normothermic machine perfusion (NMP) has emerged as a promising tool to not only improve *ex vivo* organ assessment, but also extend preservation time and provide an opportunity for organ reconditioning before transplant. Attempts to optimize NMP protocols are ongoing, and in this issue of *Kidney360*, Stone *et al.* use a porcine paired kidney analysis that demonstrates promising results using a novel, modified NMP protocol.^2^

Before modern preservation strategies, organs were stored and preserved exclusively with static cold storage (SCS), which is simple and low cost. As an alternative to SCS, hypothermic machine perfusion (HMP), which provides circulatory support for the organ before transplant, was shown in an international randomized trial to reduce DGF and improve short-term graft survival compared with SCS.^3^ Long-term follow-up data from that cohort were recently reported, demonstrating sustained improved graft outcomes that extend out to 10 years post-transplant.^4^ However, since the hypothermic conditions render the kidney metabolically inactive, *ex vivo* viability assessment of HMP kidneys is limited to assessment of perfusion parameters (*e.g*., flow, resistance), which have relatively poor predictive value.

As a contrast to HMP, NMP provides *ex vivo* oxygenated circulatory support at normal (or near-normal) body temperatures, providing an environment that restores physiologic metabolic and cellular function, allowing the organ to rehabilitate and be functionally evaluated. Kidney NMP provides access to the kidney, the perfusate, and the urine produced by the kidney *ex situ*. As a result, the kidney can be assessed beyond perfusion parameters, incorporating quantitation of urine volume, measurement of metabolic parameters, assessment of urine or perfusate injury biomarkers, and direct measures of glomerular filtration.

By evaluating a variety of functional parameters during NMP, viability determination for marginal kidneys may be performed, ideally identifying kidneys with reassuring NMP profiles that may be suitable for transplant. As such, kidney NMP has the potential to improve allocation and transplantation of hard-to-place kidneys, reduce discards of viable organs, while still maintaining acceptable post-transplant outcomes. The clinical use of NMP has experienced great success in non–kidney solid organ transplantation, particularly in liver transplantation where its application has improved early graft outcomes, extended preservation time, reconditioned marginal grafts, and reduced discard rates.^5,6^ Owing to the complexity of kidney perfusion and metabolic function, as well as production of urine *ex situ*, kidney NMP has yet to achieve routine incorporation into clinical practice, although its potential has been demonstrated in preclinical models and a growing body of clinical data.

Recent pioneering clinical trials explored the clinical application of kidney NMP. The first, from Hosgood *et al.*,^7^ which was a follow-up of their earlier pilot work,^8^ employed a short, 1-hour period of NMP at the transplant center which followed a period of conventional SCS. The authors demonstrated that among 170 kidneys treated with NMP and subsequently transplanted, safe and feasible transplantation could be performed, although no differences in DGF or 1-year graft outcomes were observed when compared with kidneys preserved with SCS alone.

The Normothermic Kidney Perfusion Phase 1 trial of NMP kidney transplant used longer perfusion times and demonstrated comparable clinical outcomes despite longer preservation time.^9^ In addition, the study provides compelling correlation of perfusate glutathione serum transferase-Pi delta as a potential biomarker for prognostication of post-transplant renal function.

In the United States, a centralized center for machine perfusion at 35°C (subnormothermic acellular machine perfusion) evaluated a series of parameters including the Hosgood Quality Assessment Score, comprised of renal blood flow, urine output, and macroscopic appearance.^10^ This protocol allowed for transplant of 142 kidneys (from 158 kidneys subject to perfusion after screening 596 unallocated kidneys), but the clinical outcomes of these transplant recipients are not yet available.

Key questions remain about the optimal NMP model and approach including perfusion duration (dose), technical aspects of the circuit design, handling of the *ex situ* urine output, and importantly, the optimal perfusate components. Stone *et al.* demonstrate the potential for improved donor preservation related to modifications in the NMP system.^2^ Their study used a porcine paired kidney model (*n*=5) to test a novel physiological NMP protocol (pNMP) for an extended 12-hour duration, compared with an existing clinical NMP (cNMP) protocol used in the United Kingdom based on the pivotal study from Hosgood *et al*.^7^ Both protocols used autologous red blood cells for oxygen delivery, and both used a creatinine bolus to allow functional assessments of the kidneys; however, there were key protocol differences. In addition to variations in the machine/circuit, the pNMP protocol provided osmotic support with albumin, vasoactive support with verapamil+epoprostenol (compared with prostacyclin in cNMP), targeted slightly higher arterial perfusion pressures, and replaced discarded urine output 1:1 with Ringer's solution.

The study showed that kidneys supported with pNMP had more favorable perfusion parameters, improved markers of functional aerobic metabolism, and lower levels of neutrophil gelatinase–associated lipocalin, a marker of tubular injury. Notably, while polyuria was observed in the cNMP kidneys, pNMP kidneys maintained stable urine output within a normal physiologic range. Finally, kidneys from the pNMP circuit had superior clinical assessment scores with less tubular necrosis than the kidneys preserved with the cNMP protocol. Although none of these kidneys were transplanted, the notable difference in tissue preservation is significant, setting the stage for this protocol to be translated into clinical trials.

As the promise of routine clinical application of kidney NMP inches closer, several questions still must remain. First, there is need for multicenter randomized trials to not only further confirm safety of NMP in kidney transplantation but also demonstrate impact on clinical outcomes including DGF, post-transplant graft function and survival, and reduction in organ discards. Second, for NMP to be successfully implemented for pre-transplant viability determination, perfusate biomarkers (and other parameters) that best correlate with DGF and graft outcomes need to be identified, refined, and validated. Third, additional preclinical studies are needed to determine the optimal perfusate composition and evaluate the role for NMP pharmacologic intervention that may allow for organ modification that reduces the harm of ischemia-reperfusion injury or immune-related injury.

Finally, it will be necessary to determine the donor kidneys that are best suited for NMP, along with the optimal deployment strategy. Currently, most kidney NMP clinical experiences use a back to base approach whereby a kidney is initially stored and transported using SCS or HMP, followed by a short period of NMP upon arrival at the transplant center. An alternative is the use of a centralized service in which the kidney is transported to a centralized NMP facility, placed on a perfusion circuit for the purpose of organ rehabilitation and viability assessment, and then transported to the transplant hospital through SCS or HMP. An additional potential option is the device-to-donor approach that initiates NMP shortly after procurement, and the organ is transported to the transplant hospital on NMP. The potential benefit of this approach is the true reduction in cold ischemic time, but such a strategy would require development of portable devices and require perfusion technical experts to accompany the organ during transport.

Kidney NMP represents a pivotal technological advancement with potential to help bridge the gap between kidney donor supply and demand. The accumulation of preclinical and clinical data suggests that the time has come for prospective trials powered to detect clinical end points. These studies are now on the horizon and will help define the role of NMP in kidney transplantation.
